# Nomogram model combined thrombelastography for venous thromboembolism risk in patients undergoing lung cancer surgery

**DOI:** 10.3389/fphys.2023.1242132

**Published:** 2023-12-14

**Authors:** Da Qin, Hongfei Cai, Qing Liu, Tianyu Lu, Ze Tang, Yuhang Shang, Youbin Cui, Rui Wang

**Affiliations:** ^1^ Department of Thoracic Surgery, The First Hospital of Jilin University, Changchun, China; ^2^ Organ Transplantation Center, The First Hospital of Jilin University, Changchun, China

**Keywords:** venous thrombosis, lung, tumor models, surgery, decision curve analysis

## Abstract

**Background:** The aim of this study was to develop a nomogram model in combination with thromboelastography (TEG) to predict the development of venous thromboembolism (VTE) after lung cancer surgery.

**Methods:** The data of 502 patients who underwent surgical treatment for lung cancer from December 2020 to December 2022 were retrospectively analyzed. Patients were then randomized into training and validation groups. Univariate and multivariate logistic regression analyses were carried out in the training group and independent risk factors were included in the nomogram to construct risk prediction models. The predictive capability of the model was assessed by the consistency index (C-index), receiver operating characteristic curves (ROC), the calibration plot and decision curve analysis (DCA).

**Results:** The nomogram risk prediction model comprised of the following five independent risk factors: age, operation time, forced expiratory volume in one second and postoperative TEG parameters k value(K) and reaction time(R). The nomogram model demonstrated better predictive power than the modified Caprini model, with the C-index being greater. The calibration curve verified the consistency of nomogram between the two groups. Furthermore, DCA demonstrated the clinical value and potential for practical application of the nomogram.

**Conclusion:** This study is the first to combine TEG and clinical risk factors to construct a nomogram to predict the occurrence of VTE in patients after lung cancer surgery. This model provides a simple and user-friendly method to assess the probability of VTE in postoperative lung cancer patients, enabling clinicians to develop individualized preventive anticoagulation strategies to reduce the incidence of such complications.

## Introduction

Venous thromboembolism (VTE), including pulmonary embolism (PE) and deep vein thrombosis (DVT), commonly co-occur with malignant tumor patients, while surgical treatment also increases the risk of VTE (Khorana, 2010). And as the most common type of cancer that poses a serious threat to human health, lung cancer has been proved by multiple studies to increase the risk of VTE (De Martino et al., 2012), with a higher postoperative VTE incidence rate of up to 12.1%–26% (Ziomek et al., 1993; Agzarian et al., 2016). Postoperative VTE is mainly due to DVT, which can be divided into the following types: central DVT is iliac-femoral vein thrombosis, which is easily treated in time because it is located in the main veins of the lower limbs and is prone to clinical symptoms such as pain and lower limb oedema; peripheral DVT refers to deep vein thrombosis below the femoral vein, which mainly includes popliteal and intermuscular veins with a relatively high incidence rate in clinical practice (Kerr et al., 1990). Among patients with VTE after lung cancer surgery, muscular calf vein thrombosis (MCVT) accounts for the vast majority and is often asymptomatic, which can easily be overlooked in clinical practice (Tian et al., 2018). PE is one of the serious complications that increases mortality after pulmonary resection, with studies showing that 10% of patients with DVT can develop PE (Geerts et al., 2001). VTE therefore needs to be given adequate attention in clinical work-up, and different levels of prophylaxis are needed for high-risk patients.

Several models exist for VTE risk assessment in different populations, with the Caprini model being the most applied in surgical patients (Hachey et al., 2016) and the modified Caprini model also being widely used in the perioperative period in thoracic surgery with good stratification capabilities (Hachey et al., 2016; Ke et al., 2022). However, the above models were developed based on data from western populations, and in recent years, low-dose CT (LDCT) screening has greatly increased the incidence of early-stage lung cancer at clinical stage T1-2N0M0, particularly in non-smoking Asian women (Howington et al., 2013), which has led to significant changes in the characteristics of the current thoracic surgery lung cancer population. Thus, the ability of the above models to identify risk in the existing lung cancer population is unknown. In summary, it is important to search for new predictive biomarkers for the current lung cancer patient population to establish an efficient prediction model.

Thrombelastography (TEG), invented by Hartert, is specifically designed to assess the overall coagulation dynamics and strength in whole blood, and has been successfully used for clinical detection of hypercoagulable states (McCrath et al., 2005; Bischof et al., 2010; Toukh et al., 2014). It has been increasingly used to assess postoperative hypercoagulability in a variety of surgical procedures and therefore holds promise for predicting the development of VTE, but its potential value in predicting the risk of postoperative VTE in patients with lung cancer remains unclear.

A nomogram is an intuitive graphical tool for rapidly predicting the risk probability of clinical events (Iasonos et al., 2008). It converts the traditional regression model into a visual risk assessment for each patient, making it practical for clinical applications. It has been widely used in studies on cancer patient prognosis and postoperative VTE prediction (Lv et al., 2021; Zhang et al., 2021; Jiang et al., 2022; Liang et al., 2022). Therefore, this study combines TEG and clinical VTE risk factors to develop a nomogram to predict the probability of postoperative VTE in lung cancer patients, in order to guide clinicians to accurately identify patients at high risk of postoperative VTE.

## Materials and methods

### Patient enrollment

A total of 652 patients with clinically diagnosed pulmonary occupying lesions in the Department of Thoracic Surgery from December 2020 to December 2022 were included consecutively. 502 cases met the inclusion and exclusion criteria of this study. The inclusion criteria were as follows: 1) pre-operative routine assessment of cardiopulmonary function and all organs, no contraindications to surgery; 2) perioperative pathology confirmed primary lung cancer; 3) underwent surgical treatment for lung cancer at our center’s thoracic surgery department with a complete clinical profile. Exclusion criteria were as follows: 1) severe abnormalities in coagulation or serious hepatic or renal impairment on post-admission examination; 2) receiving anticoagulant drugs, or hormonal drugs that affect coagulation, prior to admission, or have been discontinued but not for longer than the drug’s metabolism time; 3) postoperative pathology suggesting non-primary lung cancer; 4) DVT has been detected on preoperative lower extremity vascular ultrasound examination.

### Outcome and variables

This study’s outcome variable is VTE events occurring before discharge in lung cancer patients after surgery. All lung cancer patients routinely undergo lower extremity vascular ultrasound examination within 1 week before surgery and 3 days after surgery. Lower extremity vascular ultrasound examination is performed by experienced professionals in the ultrasound department or at the patient’s bedside. All patients experiencing unexplained hypoxemia, chest pain, hemoptysis and dyspnea during the perioperative period, regardless of whether lower extremity vascular ultrasound examination suggests DVT, should undergo CT pulmonary arteriography (CTPA) examination to determine if pulmonary embolism was present.

The electronic health record (Hospital Information System, HIS) was used for data collection. Data were extracted from the patients’ electronic medical records, which included basic information, tumor-related conditions, surgery information and laboratory indicators. Basic information includes age, gender, body mass index, history of hypertension, diabetes, stroke, cardiac stent placement, pre-operative ECG for abnormalities, anticoagulant use, smoking and alcohol consumption, preoperative pulmonary function and blood gas analysis, history of varicose veins, history of previous surgery and tumors. Tumor-related conditions includes preoperative imaging appearance, histological type of lung cancer, lung cancer TNM staging (8th edition of AJCC), neoadjuvant therapy status, postoperative pathological indication of lymph node metastasis, and postoperative pathological maximum diameter. Surgery information includes choice of surgical approach, number of lymph node dissections, lesion location, duration of surgery, intraoperative bleeding volume, whether transfusion is needed during the perioperative period, whether PICC was implanted, and length of hospital stay after surgery. Laboratory indicators include pre-operative coagulation, lipids, homocysteine, erythrocyte sedimentation rate, glycosylated hemoglobin, pre- and post-operative blood count, liver function, ions, TEG parameters and the resulting NLR (Neutrophil/Lymphocyte), PLR (Platelet count/Lymphocyte). TEG parameters include Reaction Time(R), K Value (K), Alpha Angle (a°), Maximum Amplitude (MA), Estimated Percent Lysis (EPL), Clot Lysis (LY30), Coagulation Index (CI).

### Nomogram construction and validation

All patients were randomly divided into training and validation cohorts in a 7:3 ratio to construct and validate the nomogram. Independent risk factors for the development of VTE postoperatively were determined by univariate and multivariate logistic regression analysis. Statistically significant indexes in the univariate logistic analysis and clinically significant variables were included in the multivariate logistic analysis, thus independent risk factors for postoperative VTE were determined by statistical analysis. A nomogram was then constructed using the RMS package in the R software (R 4.2.3) to visualize and score the individual risk probability of developing VTE after surgery in lung cancer patients.

The accuracy of the model to discriminate between VTE and non-VTE was assessed using the receiver operating characteristic curve (ROC) and the consistency index (C-index). The area under the curve (AUC) of the ROC was used to quantitatively evaluate the ability of the nomogram to discriminate between the occurrence of VTE in lung cancer patients after surgery. Possible values of the AUC ranged from 0.5 (no better discrimination than chance) to 1.0 (full discrimination). Calibration curve was used to show the fit between the predicted and actual probabilities estimated from the nomogram. Finally, the value of the nomogram in guiding clinical decision-making for postoperative thromboprophylaxis in lung cancer is illustrated by decision curve analysis (DCA).

### Statistical analyses

All statistical analyses were performed using IBM SPSS Statistics (26.0) and R software (4.2.3). Normally distributed measures were expressed as mean ± standard deviation (Mean ± SD) and compared between groups using the Student’s t-test; measures that did not obey a normal distribution were expressed as median (interquartile spacing) and compared between groups using the non-parametric test; Categorical variables were expressed as frequencies (percentages) and compared between groups using the chi-square test or Fisher’s exact test. ROC curve, calibration curve and DCA were performed using the “proc,” “resource selection” and “rmda” packages. *p*-value <0.05 was considered a statistically significant difference.

## Results

### Patient characteristics

**FIGURE 1:**
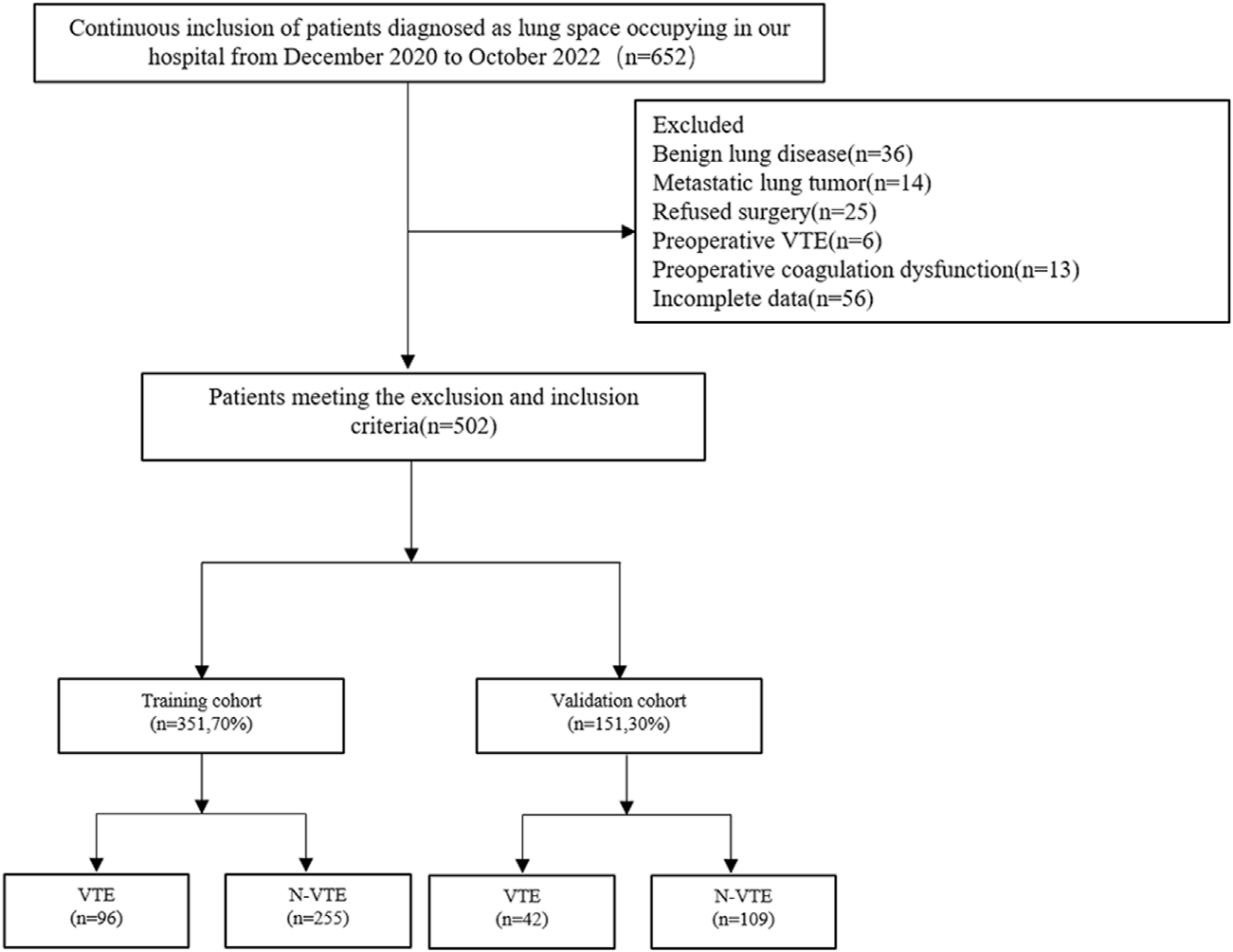
Include exclusion flow chart. VTE, venous thromboembolism.

A total of 502 patients were finally enrolled in the study, and the screening process is shown in Figure 1. Among them, 138 patients developed VTE after surgery, all of whom were diagnosed with DVT, and 6 cases had a concomitant pulmonary embolism. All patients were randomly assigned to the training and validation groups at a ratio of 7:3, and there were no significant differences in the characteristic parameters between the two groups, indicating comparability. The comparison of basic information, tumor-related conditions, surgical information, and laboratory indicators between the two groups of patients is shown in Table 1.

**TABLE 1:** Demographic and clinical characteristics of patients in training and validation cohorts.

Variable	Total (N = 502)	Training cohort (N = 351)	Validation cohort (N = 151)	t/χ2	*p*
VTE	138	96 (69.6%)	42 (30.4%)	0.011	0.915
Age	56.99 ± 10.28	57.06 ± 10.23	56.83 ± 10.42	0.222	0.824
Sex				0.087	0.768
Male	171	121 (70.8%)	50 (29.2%)		
Female	331	230 (69.5%)	101 (30.5%)		
BMI(kg/m^2^)	24.18 ± 3.28	24.20 ± 3.22	24.11 ± 3.41	0.286	0.775
Hypertension				0.257	0.612
No	402	279 (69.4%)	123 (30.6%)		
Yes	100	72 (72.0%)	28 (28.0%)		
Diabetes				<0.001	0.989
No	442	309 (69.9%)	133 (30.1%)		
Yes	60	42 (70.0%)	18 (30.0%)		
Pre-operative ECG for abnormalities				0.249	0.519
No	457	321 (70.2%)	136 (29.8%)		
Yes	45	30 (66.7%)	15 (33.3%)		
Smoking history				0.292	0.589
No	390	275 (70.5%)	115 (29.5%)		
Yes	112	76 (67.9%)	36 (32.1%)		
Alcohol history				0.082	0.775
No	442	310 (70.1%)	132 (29.9%)		
Yes	60	41 (68.3%)	19 (31.7%)		
Varicose veins					1.000*
No	489	342 (69.9%)	147 (30.1%)		
Yes	13	9 (69.2%)	4 (30.8%)		
FEV1(L)	2.40 ± 0.58	2.40 ± 0.57	2.39 ± 0.58	0.131	0.896
FEV1/FVC(%)	81.35 ± 9.07	81.31 ± 9.05	81.47 ± 9.16	−0.172	0.863
Preoperative imaging appearance				0.332	0.847
GGO	48	32 (66.7%)	16 (33.3%)		
Subsolidity	429	302 (70.4%)	127 (29.6%)		
Solidity	25	17 (68.0%)	8 (32.0%)		
Histological type				0.338	0.845
AD	464	324 (69.8%)	140 (30.2%)		
SCC	20	15 (75.0%)	5 (25.0%)		
Others	18	12 (66.7%)	6 (33.3%)		
Multiple nodules on imaging				0.255	0.614
No	238	169 (71.0%)	69 (29.0%)		
Yes	264	182 (68.9%)	82 (31.1%)		
Lymph nodes positive				0.017	0.897
No	460	322 (70.0%)	138 (30.0%)		
Yes	42	29 (69.0%)	13 (31.0%)		
Pathological diameter(cm)	1.58 ± 0.98	1.58 ± 0.99	1.58 ± 0.98	−0.009	0.993
Surgical approach					0.702*
VATS	494	346 (70.0%)	148 (30.0%)		
Open	8	5 (62.5%)	3 (37.5%)		
LNR	6.68 ± 2.54	6.73 ± 2.57	6.56 ± 2.47	0.684	0.494
Operation duration	136.58 ± 46.60	135.70 ± 45.94	138.62 ± 48.20	−0.645	0.519
Bleeding volume	74.14 ± 53.59	72.62 ± 50.69	77.68 ± 59.83	−0.970	0.332
PICC implanted					1.000*
No	493	345 (70.0%)	148 (30.0%)		
Yes	9	6 (66.7%)	3 (33.3%)		
Pre-PLT	223.74 ± 54.72	223.77 ± 55.15	221.34 ± 53.83	0.642	0.521
Pre-TT	17.34 ± 1.07	17.34 ± 1.06	17.33 ± 1.09	0.188	0.851
Pre- APTT	26.60 ± 2.39	26.57 ± 2.39	26.65 ± 2.40	−0.362	0.717
Pre- PT	11.20 ± 0.69	11.19 ± 0.69	11.24 ± 0.68	−0.660	0.509
Pre-Fbg	2.76 ± 0.71	2.77 ± 0.72	2.74 ± 0.69	0.514	0.608
Pre-NLR	1.85 ± 1.00	1.86 ± 1.00	1.83 ± 1.02	0.223	0.824
Pre-PLR	132.05 ± 84.44	132.85 ± 83.27	130.19 ± 87.35	0.322	0.748
TC	4.94 ± 0.96	4.94 ± 0.99	4.93 ± 0.91	0.087	0.930
TG	1.57 ± 0.93	1.57 ± 0.92	1.58 ± 0.96	−0.051	0.960
HDL-C	1.19 ± 0.31	1.19 ± 0.30	1.58 ± 0.96	−0.308	0.758
LDL-C	3.15 ± 0.73	3.16 ± 0.75	3.14 ± 0.69	0.174	0.862
HCY	10.05 ± 4.3	10.04 ± 4.35	10.07 ± 4.21	−0.043	0.966
Pre-R	5.67 ± 0.93	5.68 ± 0.92	5.65 ± 0.94	0.319	0.750
Pre-K	1.76 ± 0.47	1.76 ± 0.47	1.74 ± 0.46	0.333	0.739
Pre- a°	64.89 ± 5.73	64.89 ± 5.78	64.90 ± 5.63	−0.009	0.993
Pre-MA	63.18 ± 5.90	63.29 ± 5.98	62.91 ± 5.71	0.591	0.555
Pre-CL	0.52 ± 1.41	0.52 ± 1.43	0.52 ± 1.36	−0.008	0.994
Post-PLT	202.47 ± 55.27	203.30 ± 55.52	200.54 ± 54.83	0.514	0.608
Post-NLR	12.91 ± 9.41	12.80 ± 9.45	13.15 ± 9.32	−0.379	0.705
Post-PLR	253.19 ± 142.7	252.25 ± 143.82	255.38 ± 140.5	−0.225	0.822
Post-R	5.24 ± 0.80	5.25 ± 0.80	5.23 ± 0.80	0.257	0.797
Post-K	1.70 ± 0.47	1.70 ± 0.47	1.70 ± 0.49	0.046	0.963
Post- a°	65.79 ± 5.07	65.74 ± 5.02	65.92 ± 5.20	−0.347	0.729
Post-MA	65.08 ± 4.87	65.06 ± 4.81	65.11 ± 5.02	−0.104	0.917
Post-CL	1.28 ± 1.16	1.27 ± 1.14	1.30 ± 1.20	−0.241	0.810

*p values were derived from Fisher Exact test.

VTE, venous thromboembolism; BMI, body mass index; ECG, electrocardiogram; FEV1, forced expiratory volume in one second; FVC, forced vital capacity; GGO, ground- glass opacity; AD, adenocarcinoma; SCC, squamous cell carcinoma; VATS, video- assisted thoracoscopic surgery; LNR, lymph node removal; PICC, peripherally inserted central catheter; Pre-, preoperative; Post-, postoperative; PLT, platelet; TT, thrombin time; APTT, activated partial thromboplastin time; PT, prothrombin time; Fbg, fibrinogen; NLR, neutrophil/lymphocyte; PLR, platelet count/lymphocyte; TC, total cholesterol; TG, triglyceride; HDL-C, high density lipoprotein- cholesterol; LDL-C, low density lipoprotein-cholesterol; HCY, homocysteine; R, reaction time; K, k value; a°, alpha angle; MA, maximum amplitude; CI, coagulation index.

### Independent risk factors for VTE in the training set

In the univariate logistic analysis of the training cohort, it was found that age (OR = 1.104, 95% CI: 1.060–1.150, *p* < 0.001), operation duration (OR = 1.016, 95% CI: 1.009–1.022, *p* < 0.001), bleeding volume (OR = 1.010, 95% CI: 1.004–1.016, *p* = 0.002), postoperative TEG parameters a°(OR = 1.256 95% CI: 1.154–1.367, *p* < 0.001), MA(OR = 1.181 95% CI: 1.096–1.273, *p* < 0.001) and CI(OR = 3.189 95% CI: 2.107–4.826, *p* < 0.001) were significantly positively correlated with postoperative VTE, while FEV1(OR = 0.301 95% CI: 0.159–0.573, *p* < 0.001), multiple nodules on imaging (OR = 0.420 95% CI: 0.230–0.767, *p* = 0.005), postoperative TEG parameters K(OR = 0.015 95% CI: 0.004–0.057, *p* < 0.001) and R (OR = 0.172 95%CI: 0.093–0.319, *p* < 0.001) were significantly negatively correlated with postoperative VTE. CI is the result of comprehensive calculation of other parameters of TEG, taking into account multicollinearity, so it is not included in the multivariate logistic analysis. Considering the small sample size, therefore, other statistically significant factors including: age, FEV1, multiple nodules on imaging, operation duration, bleeding volume, postoperative TEG parameters R, K, a°, and MA, as well as clinically significant factors including: BMI, varicose veins, and histological type were included in the multivariate analysis. It was found that age (OR = 1.082 95% CI: 1.023–1.145, *p* = 0.006), FEV1(OR = 0.350 95% CI: 0.133–0.923, *p* = 0.034), operation duration (OR = 1.017 95% CI:1.004–1.030, *p* = 0.012), postoperative TEG parameters K(OR = 0.050 95% CI:0.004–0.566, *p* = 0.016) and R (OR = 0.253 95% CI:0.104–0.615, *p* = 0.002) were independent risk factors for postoperative VTE. The above results are shown in Table 2.

**TABLE 2:** Logistic regression analysis for VTE in the training cohort.

Variable	Univariate analysis		*P*	Multivariate analysis		*P*
	OR	95% CI		OR	95% CI	
**Age**	1.104	1.060–1.150	**<0.001**	1.082	1.023–1.145	**0.006**
**Sex**						
**Male**	Ref					
**Female**	0.946	0.518–1.728	0.946			
**BMI(kg/m** ^**2**^ **)**	1.045	0.956–1.142	0.333	1.115	0.945–1.317	0.198
**Hypertension**						
**No**	Ref					
**Yes**	0.656	0.333–1.291	0.222			
**Diabetes**						
**No**	Ref					
**Yes**	0.613	0.264–1.422	0.254			
**Pre-operative ECG for abnormalities**						
**No**	Ref					
**Yes**	0.758	0.249–2.313	0.627			
**Smoking history**						
**No**	Ref					
**Yes**	0.784	0.384–1.600	0.503			
**Alcohol history**						
**No**	Ref					
**Yes**	0.795	0.325–1.947	0.616			
**Varicose veins**						
**No**	Ref			Ref		
**Yes**	0.575	0.094–3.528	0.550	0.678	0.033–14.083	0.802
**FEV1(L)**	0.301	0.159–0.573	**<0.001**	0.350	0.133–0.923	**0.034**
**FEV1/FVC(%)**	0.984	0.952–1.017	0.339			
**Preoperative imaging appearance**						
**GGO**	Ref					
**Subsolidity**	0.561	0.216–1.455	0.235			
**Solidity**	0.375	0.063–2.244	0.283			
**Histological type**						
**AD**	Ref			Ref		
**SCC**	0.266	0.058–1.227	0.090	0.459	0.041–5.193	0.530
**Others**	0.500	0.070–3.550	0.488	0.162	0.003–8.295	0.365
**Multiple nodules on imaging**						
**No**	Ref			Ref		
**Yes**	0.420	0.230–0.767	**0.005**	0.490	0.198–1.214	0.123
**Lymph nodes positive**						
**No**	Ref					
**Yes**	0.632	0.237–1.685	0.359			
**Pathological diameter(cm)**	1.029	0.755–1.402	0.857			
**Surgical approach**						
**VATS**	Ref					
**Open**	5.344	0.476–60.006	0.174			
**LNR**	1.062	0.944–1.194	0.317			
**Operation duration**	1.016	1.009–1.022	**<0.001**	1.017	1.004–1.030	**0.012**
**Bleeding volume**	1.010	1.004–1.016	**0.002**	1.003	0.991–1.016	0.593
**Pre-PLT**	0.997	0.992–1.003	0.332			
**Pre-TT**	0.812	0.604–1.090	0.166			
**Pre- APTT**	0.888	0.780–1.011	0.073			
**Pre- PT**	1.116	0.725–1.716	0.618			
**Pre-Fbg**	0.992	0.954–1.031	0.671			
**Pre-NLR**	0.902	0.649–1.255	0.541			
**Pre-PLR**	0.997	0.991–1.003	0.257			
**TC**	1.429	0.942–2.169	0.093			
**TG**	1.366	0.881–2.118	0.163			
**HDL-C**	1.511	0.397–5.752	0.545			
**LDL-C**	1.578	0.903–2.755	0.109			
**HCY**	0.962	0.911–1.016	0.160			
**Pre-R**	0.814	0.564–1.176	0.274			
**Pre-K**	0.691	0.327–1.459	0.332			
**Pre- a°**	1.028	0.972–1.088	0.333			
**Pre-MA**	0.996	0.978–1.014	0.657			
**Pre-CL**	1.104	0.884–1.378	0.382			
**Post-PLT**	0.996	0.991–1.002	0.171			
**Post-NLR**	0.984	0.945–1.024	0.415			
**Post-PLR**	0.997	0.995–1.000	0.059			
**Post-R**	0.172	0.093–0.319	**<0.001**	0.253	0.104–0.615	**0.002**
**Post-K**	0.015	0.004–0.057	**<0.001**	0.050	0.004–0.566	**0.016**
**Post- a°**	1.256	1.154–1.367	**<0.001**	0.947	0.815–1.110	0.476
**Post-MA**	1.181	1.096–1.273	**<0.001**	1.061	0.933–1.207	0.365
**Post-CL**	3.189	2.107–4.826	**<0.001**			

VTE, venous thromboembolism; BMI, body mass index; ECG, electrocardiogram; FEV1, forced expiratory volume in one second; FVC, forced vital capacity; GGO, ground- glass opacity; AD, adenocarcinoma; SCC, squamous cell carcinoma; VATS, video- assisted thoracoscopic surgery; LNR, lymph node removal; PICC, peripherally inserted central catheter; Pre-, preoperative; Post-, postoperative; PLT, platelet; TT, thrombin time; APTT, activated partial thromboplastin time; PT, prothrombin time; Fbg, fibrinogen; NLR, neutrophil/lymphocyte; PLR, platelet count/lymphocyte; TC, total cholesterol; TG, triglyceride; HDL-C, high density lipoprotein- cholesterol; LDL-C, low density lipoprotein- cholesterol; HCY, homocysteine; R, reaction time; K, k value; a°, alpha angle; MA, maximum amplitude; CI, coagulation index.

### Nomogram construction

**FIGURE 2:**
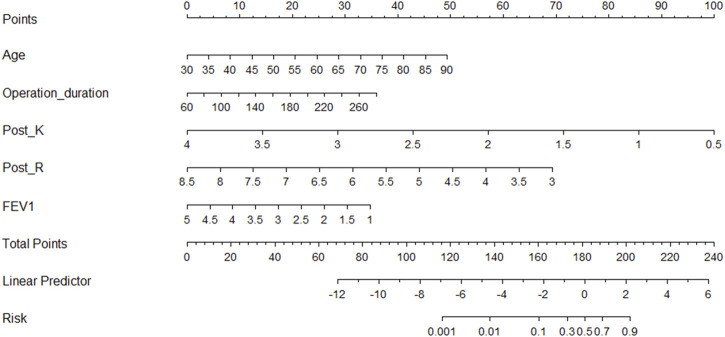
Nomogram for predicting postoperative VTE in patients with lung cancer. Post_K, postoperative TEG parameters k value; Post_R, postoperative TEG parameters reaction time; FEV1, forced expiratory volume in one second.

According to the regression coefficient of risk factors in multivariate analysis, the risk prediction model of postoperative VTE was established as follows: Logit(P) = 5.278 + 0.088×Age-0.928×FEV1+0.017×Operation duration-3.05×Post-K-1.345×Post-R. In order to facilitate the clinical application of the prediction model, the above model was visualized as nomogram to predict the possibility of postoperative VTE of lung cancer (Figure 2). The application of nomogram is as follows: locate the patient’s age on the age axis. Draw a line up to the Points axis and determine the corresponding score. Repeat the above process for other predictive variables and finally add the scores. Draw a line down the point of the corresponding total score on the total points axis to determine the VTE risk. For example, a 65-year-old patient whose operative time was 160 min, postoperative TEG K value was 1.5 min, R value was 4 min, FEV1 was 2L. The score of each variable was 29, 16, 71, and 57, respectively, with a total score of 199. The corresponding risk of postoperative VTE was 87%.

### Calibration and validation of the nomogram

**FIGURE 3:**
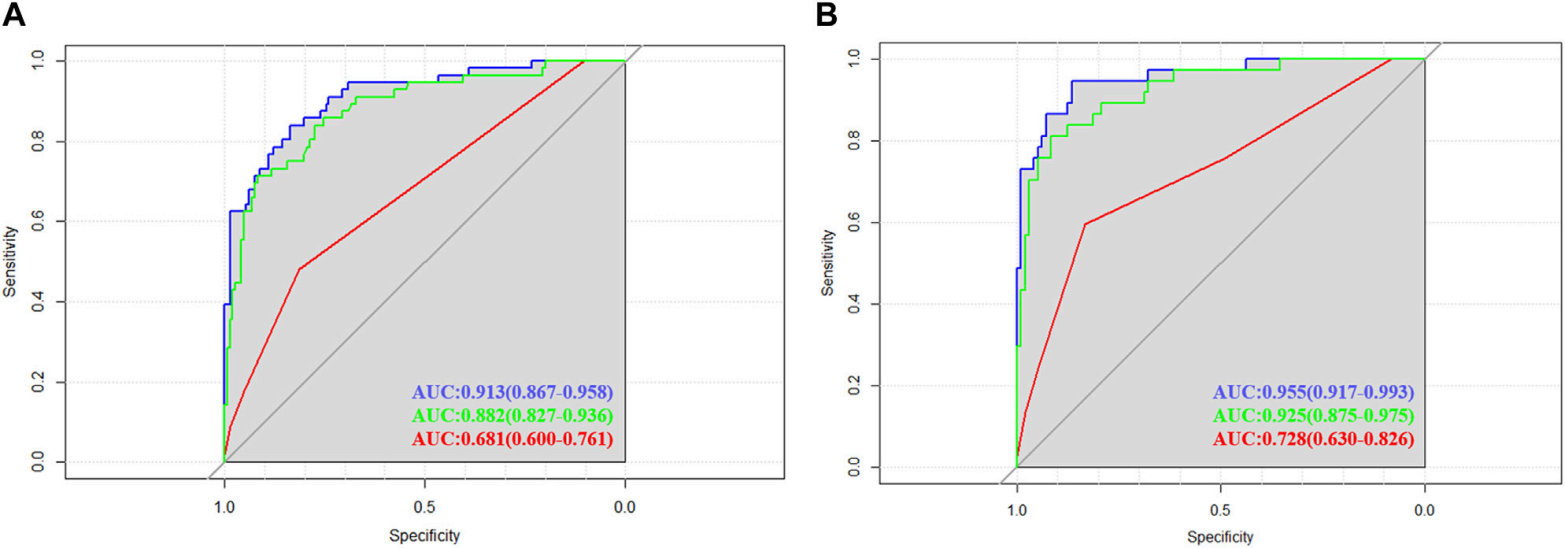
Receiver operating characteristic (ROC) curves in training and validation cohorts. **(A)** The area under the ROC curve for the nomogram (blue line), nomogram consisting of Caprini scores and TEG parameters (green line), modified Caprini model (red line) in the training cohort were 0.913 (0.867–0.958), 0.882 (0.827–0.936) and 0.681 (0.600–0.761), respectively. **(B)** The area under the ROC curve for the nomogram (blue line), nomogram consisting of Caprini scores and TEG parameters (green line), modified Caprini model (red line) in the validation cohort were 0.955 (0.917–0.993), 0.925 (0.875–0.975) and 0.728 (0.630–0.826), respectively.

**FIGURE 4:**
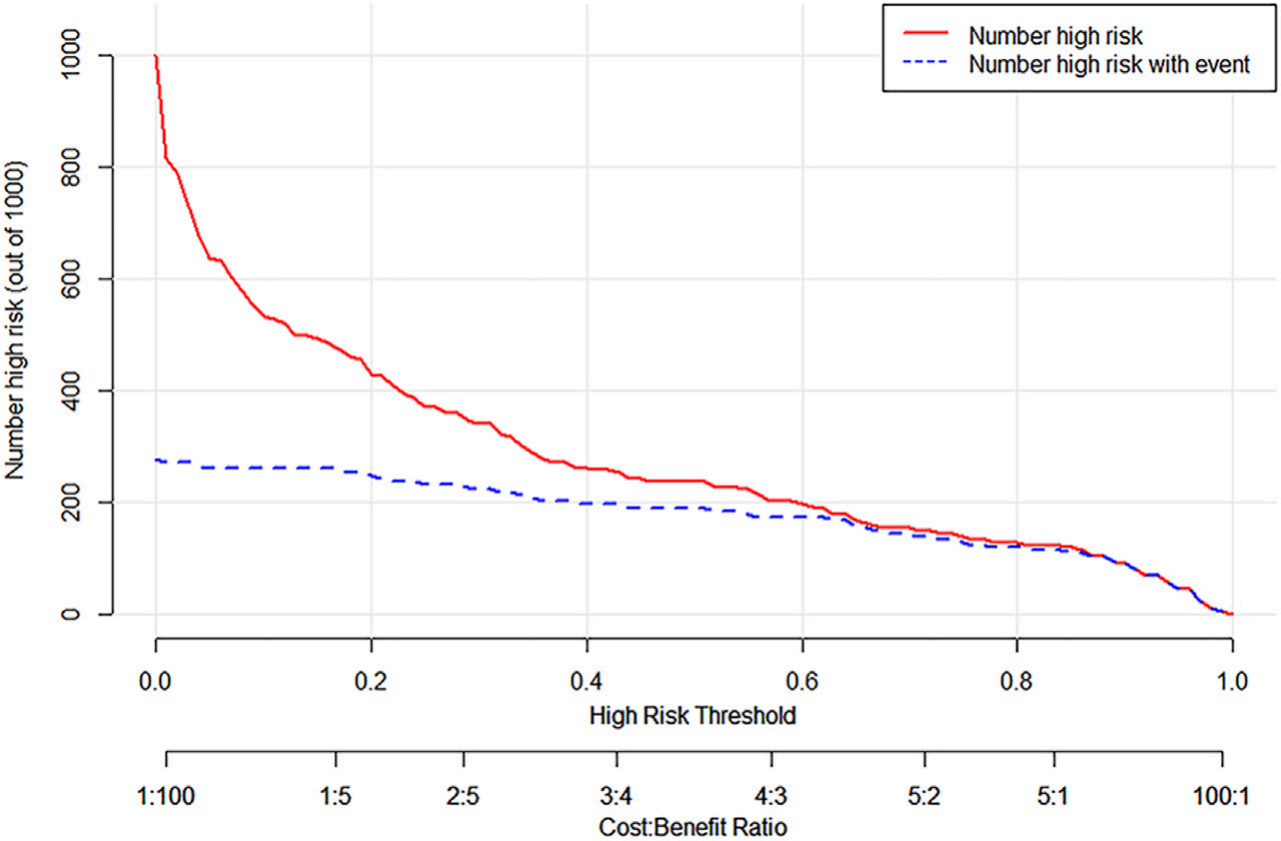
Clinical impact curve of nomogram in training cohort.

**FIGURE 5:**
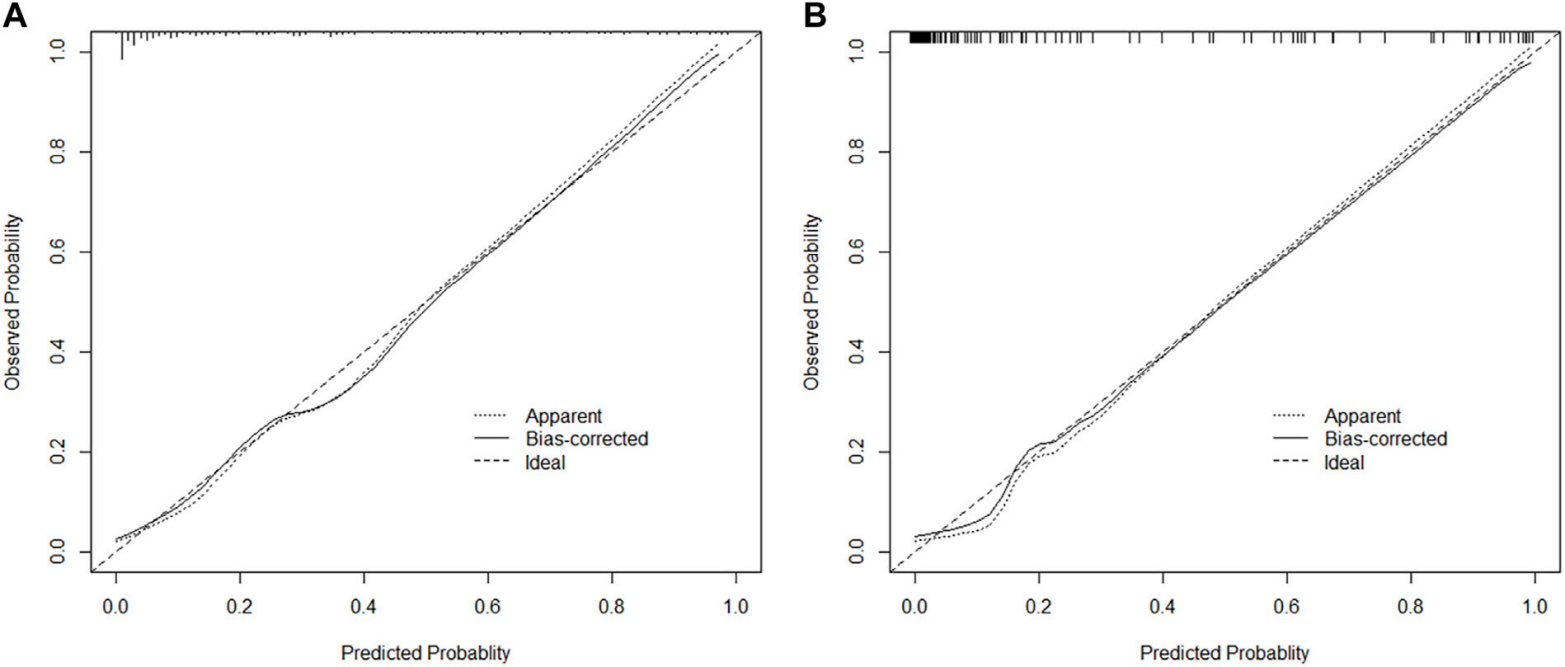
The calibration plot of nomogram in training and validation cohorts **(A,B)**. Bootstrapping method with 1000 resamples was utilized.

ROC curves were drawn in the training set and the validation set. The modified Caprini model is detailed in Supplementary Table S1. In the training set, the AUC values of nomogram and the modified Caprini model were 0.913 (0.867–0.958) and 0.681 (0.600–0.761), respectively. The results are shown in Figure 3A. In the verification set, the AUC value of nomogram is still higher than that of the modified Caprini model (0.955 (0.917–0.993) VS 0.728 (0.630–0.826)). The results are shown in Figure 3B. The study indicates that the nomogram has a better discriminative ability for predicting postoperative VTE in patients, and is higher than the existing modified Caprini model. Meanwhile, a nomogram including Caprini scores and TEG parameters was constructed (Supplementary Figure S1) and ROC curves (Figure 3) were plotted to demonstrate that the TEG parameters can improve the predictive value of the modified Caprini model. The optimal cutoff value for the ROC curve of the training set is 0.286. At this point, the sensitivity is 0.836 and the specificity is 0.839, which means that when the predicted probability is greater than 0.286, the risk of postoperative VTE is high. The clinical impact curve of the training set also proves the good prediction effect of the nomogram under this threshold (Figure 4). Meanwhile, utilizing the calibration curve, the consistency between the predicted probability and the true probability of the nomogram was demonstrated in both the training and validation sets. The C-index values are 0.913 and 0.955 respectively. Hosmer-Lemeshow goodness-of-fit test was applied to the training (X-squared = 11.569, *p* = 0.172) and validation sets (X-squared = 5.207, *p* = 0.735), and the *p* values were both greater than 0.05, indicating a good fit. The results are shown in Figures 5A, B. Considering age as an important risk factor for postoperative VTE, the significance of TEG parameters under different age stratification was explored. The results (Supplementary Table S2) suggest that age influences TEG parameters, but still different TEG parameters are independent risk factors for postoperative VTE formation under different stratifications, further demonstrating the validity of TEG for predicting postoperative VTE.

### Clinical use

**FIGURE 6:**
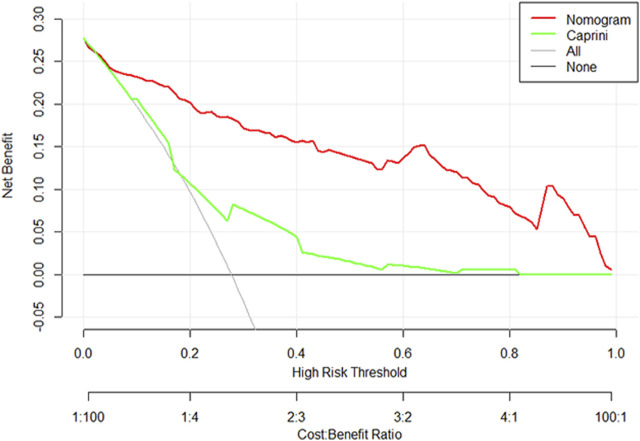
Decision curve analysis of nomogram and the modified Caprini model.

The DCA curve of the training set is shown in Figure 6. The DCA curve shows that nomogram is more beneficial in predicting the occurrence of postoperative VTE than all treatment and non-treatment strategies.

## Discussion

Lung cancer has the highest risk of death compared to other cancers and is also the second most prevalent cancer in the world’s population (Sung et al., 2021). Surgery remains an important part of lung cancer treatment today (Howington et al., 2013; Sher et al., 2015). VTE, one of the most common complications after lung cancer surgery, has a devastating impact on patient follow-up and increases the risk of postoperative mortality (Corrales-Rodriguez and Blais, 2012), which may be mainly related to subendothelial collagen exposure, hypercoagulability and reduced activity due to postoperative patient pain (Pastori et al., 2023). So, it is important to investigate the risk factors for the development of VTE in postoperative lung cancer patients and to develop a risk prediction model. TEG can be an important way to assess coagulation in perioperative thoracic patients and has the potential to predict the development of postoperative VTE in lung cancer patients. Therefore, in this study, TEG parameters were incorporated into the nomogram to predict the occurrence of VTE after lung cancer surgery. To our best knowledge, this is the first to combine TEG with clinical risk factors to construct nomogram to predict the formation of VTE after lung cancer operation. The training and validation sets were also used to verify the predictive ability and consistency of the model.

The nomogram developed for this study includes the following factors: age, operation duration, FEV1, postoperative TEG K and R. The model has a C-index of 0.913, which has shown accurate predictive power and has been internally validated to demonstrate consistency of results. Several studies have demonstrated the validity of the nomogram for predicting the occurrence of VTE in cancer patients (Li J. et al., 2021; Zhang et al., 2021; Zhang et al., 2022a; Zhang et al., 2022b; Jin et al., 2022; Li et al., 2022; Wang et al., 2022; Cai et al., 2023; Lei et al., 2023). In the study of VTE in lung cancer patients, the inclusion of subjects was focused on patients with lung cancer treated with internal therapy, and the inclusion of risk factors included genetic mutations regarding targeted therapy, therapeutic use, and the systemic immune-inflammation index, etc (Zhang et al., 2022b; Li et al., 2022; Lei et al., 2023). Cai et al. (2023) included patients with stage IA non-small cell lung cancer to establish a nomogram for the occurrence of postoperative VTE, which included three variables: age, preoperative D-dimer, and intermuscular vein dilation, with a C-index of 0.832.The different inclusion parameters compared to the present study were mainly due to the inclusion of heterogeneity of the population. Li Y. et al. (2021) developed a nomogram to predict the occurrence of pulmonary thromboembolism after lung cancer surgery, including five variables: age, BMI, operation time, the serum level of CA153 before surgery and abnormal results of CUS before surgery, which could effectively predict the risk of pulmonary embolism.

Smoking, obesity, hypertension, diabetes mellitus, dyslipidemia and elevated homocysteine have all been shown to be risk factors associated with arterial thromboembolism (Noumegni et al., 2021). In patients with major cardiovascular risk factors, the risk of arterial thrombosis is likely to be associated with an inflammatory response and a hypercoagulable state (Yu et al., 2019; Moik and Ay, 2022). Both inflammation and hypercoagulability may contribute to VTE events in these patients (Prandoni et al., 2003; Prandoni et al., 2006). This study therefore examines the impact of these risk factors associated with arterial thromboembolism on VTE. In the general data analysis, there were no statistical differences between the VTE and non-VTE groups in terms of BMI, comorbidities such as hypertension and diabetes, smoking and alcohol consumption (*p* > 0.05). In the laboratory analysis, there were no statistical differences between the two groups in terms of lipid parameters: cholesterol, triglycerides, high-density lipoprotein, low-density lipoprotein and homocysteine. Preliminarily, the risk factors associated with arterial thromboembolism did not influence the formation of VTE in patients with postoperative lung cancer.

Several studies have shown that the inflammatory response in cancer patients produces large amounts of cytokines and inflammatory mediators to stimulate the coagulation response and ultimately promote thrombosis (Preston et al., 2019). NLR (Go et al., 2014) and PLR (Yang and Liu, 2015) in peripheral blood can respond to the immune-inflammatory state of the body and have been reported to be associated with VTE formation in cancer. In this study, the inclusion of preoperative and postoperative NLR and PLR in a univariate analysis of postoperative VTE formation in lung cancer was found to be statistically insignificant, which differs from the results obtained by Zhang et al. (2022a). It may be related to the different lung cancer stages of the included subjects.

Age is a well-recognized influencing factor for VTE and this study also confirms its independent influence on the development of VTE in lung cancer patients after surgery. The risk of VTE after lung cancer becomes 1.082 times greater for each additional year of age of the patient, which is consistent with the findings of other studies on postoperative lung cancer (Thomas et al., 2018; Tian et al., 2018; Song et al., 2019). Thomas et al. (2018) showed that the risk of postoperative VTE was 2.2 times higher in patients aged 65–80 years than in those younger than 65 years.

Multiple studies have shown that operation duration affects the incidence of postoperative VTE (Kim et al., 2015; Cui et al., 2020). This was also demonstrated in this study, with the risk of VTE becoming 1.7 times greater for every 100 min longer the patient’s operative time. Prolonged operative time can result in increased intraoperative braking time, and the lateral position required for thoracoscopic lung cancer surgery further increases the risk of postoperative VTE formation. In addition, the use of narcotic drugs, the varying degrees of injury caused by manual dissection during intraoperative thoracic adhesions, and the freeing and blocking of vital vessels all increase the risk of VTE.

FEV1 is the volume of gas exhaled in the first second of maximal expiration and a common indicator of asthma as well as COPD. The present study demonstrated that FEV1 was an independent risk factor for the development of VTE in patients with postoperative lung cancer. Wang et al. (2019) showed that the results of a multivariate analysis including FEV1 after manual exclusion of age showed that FEV1 was an independent risk factor for the development of VTE after lung cancer surgery (OR = 0.278; 95% CI: 0.145–0.532), which is consistent with the results of the study. The mechanism affecting the development of VTE is the same as in COPD and may be associated with thrombosis stimulated by vascular epithelial damage due to chronic hypoxia.

A hypercoagulable state of blood in oncology patients may be an early manifestation of VTE before it occurs. Conventional coagulation tests (APTT, PT, TT, etc.) show the timing of fibrin formation through the intrinsic and extrinsic pathways of the coagulation cascade. However, the indicators they reflect in plasma limit their relevance to overall dynamic clot formation in whole blood (Eckman et al., 2003). In contrast, TEG reflects the whole process from initial thrombin generation to fibrin chain formation to fibrinolysis (Mallett and Cox, 1992). Since it was first described in 1948, TEG has been successfully used in different clinical areas (Whitten and Greilich, 2000). The greatest use has been in guiding transfusion strategies for surgical patients, such as *in situ* liver transplantation and extracorporeal circulation, where there is a risk of major bleeding (Kang, 1995; Spiess et al., 1995). In recent years, the use of TEG in drug monitoring and patient screening has led to a renewed interest in this technique (Davies et al., 2015; Artang et al., 2022). In this study, K and R of TEG were found to be independent risk factors for the development of VTE postoperatively and contributed the most in the nomogram. This shows the predictive role of TEG for postoperative VTE and improves the accuracy of the prediction model.

A variety of measures have been used clinically to assess the risk of developing VTE in cancer. In recent years, foreign thoracic surgeons have mostly used the modified Caprini model, which simplifies the risk classification into low risk (0–4 points), intermediate risk (5–8 points) and high risk (≥9 points) and is considered to be more applicable to patients with thoracic malignancies (Hachey et al., 2016; Ke et al., 2022). In this study, comparing the C-index of the nomogram with the modified Caprini model, the nomogram was found to have a higher clinical predictive value, its main possibility being the incorporation of TEG parameters with high specificity and the refinement of clinical risk factors more relevant to postoperative VTE in lung cancer.

The present study still has some limitations. Firstly, there is an unavoidable selection bias in single-center retrospective studies, which reduces the prevalence of the model. Secondly, the sample sizes of both the training and validation sets were small and the statistics may be biased. Thirdly, we lack external validation, especially from different races around the globe. Finally, studies have shown that postoperative VTE mostly occurs after hospital discharge (Thomas et al., 2018), whereas the above-mentioned patients were not included in this study at the end of follow-up until hospital discharge, which may lead to bias in the incidence of postoperative VTE.

## Conclusion

In this study, age, operation time, FEV1 and postoperative TEG parameters K and R were derived as independent risk factors for the development of VTE in lung cancer patients after surgery. The above parameters were incorporated into the nomogram risk prediction model, showing a higher predictive power than existing accepted models, which could guide individualized preventive anticoagulation therapy in lung cancer patients after surgery. Next, multicenter clinical data are needed to validate the applicability of the model.

## Data Availability

The raw data supporting the conclusion of this article will be made available by the authors, without undue reservation.
