# Supplementary material for: Nomogram model combined thrombelastography for venous thromboembolism risk in patients undergoing lung cancer surgery
Source: Front Physiol. 2023 Dec 14;14:1242132. doi: 10.3389/fphys.2023.1242132 (PMC10757630; doi:10.3389/fphys.2023.1242132)
Supplement: Supplementary file 1 [file Table1.DOCX]

| Categorization | Caprini risk factor | Caprini score |
| --- | --- | --- |
| General information | Age 40-59 (y) | 1 |
|  | Age 60-74 (y) | 2 |
|  | Age≥75 (y) | 3 |
|  | Body mass index≥30 (kg/m2 ) | 1 |
|  | Abnormal pulmonary function | 1 |
|  | Swollen legs (current) | 1 |
|  | Confined to bed (>72 h) | 2 |
| Past history | History of inflammatory bowel disease | 1 |
|  | History of prior major surgery (﹤1 mo) | 1 |
|  | History of VTE | 3 |
|  | Family history of VTE | 3 |
| Comorbidities | Acute myocardial infarction (<1 mo) | 1 |
|  | Congestive heart failure (<1 mo) | 1 |
|  | Complications of pregnancy | 1 |
|  | Sepsis (﹤1 mo) | 1 |
|  | Serious acute lung disease (<1 mo) | 1 |
|  | Varicose veins | 1 |
|  | Present cancer | 2 |
|  | Prior cancer, except nonmelanoma skin | 2 |
|  | Acute spinal cord injury (<1 mo) | 5 |
| Current treatment | Oral contraceptive use or HRT | 1 |
|  | Central venous access | 2 |
|  | Major open surgery (≥45 min) | 2 |
|  | Chemotherapy | 3 |
|  | Major surgery≥6 h | 5 |
| Immune markers | Positive anticardiolipin antibody | 3 |
|  | Positive Lupus anticoagulant | 3 |

Table S1 The modified Caprini model.

VTE, Venous thromboembolism; HRT, hormone replacement therapy; 0~ 4 is classified as low risk, 5-8 as intermediate risk, and ≥9 as high risk.
