# Supplementary material for: Nomogram model combined thrombelastography for venous thromboembolism risk in patients undergoing lung cancer surgery
Source: Front Physiol. 2023 Dec 14;14:1242132. doi: 10.3389/fphys.2023.1242132 (PMC10757630; doi:10.3389/fphys.2023.1242132)
Supplement: Supplementary file 3 [file Table2.DOCX]

| **Age** | **TEG** | **OR** | **95%CI** | ***P*** |
| --- | --- | --- | --- | --- |
| **<60 years** |  |  |  |  |
|  | **Post-R** | 0.503 | 0.149-1.700 | 0.269 |
|  | **Post-K** | 0.035 | 0.005-0.786 | **0.032** |
|  | **Post- a°** | 0.870 | 0.707-1.071 | 0.188 |
|  | **Post-MA** | 1.085 | 0.918-1.282 | 0.340 |
| **≥60 years** |  |  |  |  |
|  | **Post-R** | 0.020 | 0.002-0.231 | **0.002** |
|  | **Post-K** | 0.010 | 0.006-2.705 | 0.107 |
|  | **Post- a°** | 1.346 | 0.852-2.125 | 0.203 |
|  | **Post-MA** | 0.861 | 0.653-1.135 | 0.289 |

Table S2 Association between TEG and postoperative VTE in age stratification.

TEG, thrombelastography; R, reaction time; K, k value; a°, alpha angle; MA, maximum amplitude.
